# Carboplatin plus pemetrexed versus pemetrexed alone in advanced thymoma and thymic carcinoma: a retrospective cohort study

**DOI:** 10.3389/fonc.2026.1795920

**Published:** 2026-04-22

**Authors:** Benjamin A. Bleiberg, Lauren Reed-Guy, Roger B. Cohen, Charu Aggarwal, Corey J. Langer, Aditi P. Singh, Melina E. Marmarelis

**Affiliations:** 1 Department of Hematology-Oncology, Division of Internal Medicine, University of Pennsylvania, Philadelphia, PA, United States; 2 Penn Center for Cancer Care Innovation, University of Pennsylvania, Philadelphia, PA, United States

**Keywords:** carboplatin, chemotherapy, clinical outcomes, pemetrexed, retrospective study, thymic carcinoma, thymoma

## Abstract

**Introduction:**

Pemetrexed is approved as a single agent for advanced and metastatic thymoma and thymic carcinoma. In practice, pemetrexed is often given with carboplatin, but the safety and effectiveness of this combination has not been established in either of these rare diseases.

**Methods:**

We identified patients with unresectable, recurrent, or metastatic thymoma or thymic carcinoma who were treated with carboplatin/pemetrexed or pemetrexed alone from 2006-2024. Time to disease progression and objective response were determined retrospectively using RECIST 1.1. Median progression-free survival (mPFS) and median overall survival (mOS) were estimated using Kaplan-Meier methodology.

**Results:**

We identified 24 patients (14 thymoma; 10 thymic carcinoma), including 8 treated with carboplatin/pemetrexed and 16 with pemetrexed alone. For our combined cohort, mPFS was not reached (NR) with carboplatin/pemetrexed vs. 14.7m with pemetrexed (p=0.27). Among patients with thymoma, mPFS with carboplatin/pemetrexed was NR vs. 15.6m for pemetrexed (p=0.09), while among patients with thymic carcinoma mPFS was 10.9m vs. 14.7m (p=0.88), respectively. At a median follow-up of 49.0m (37m for carboplatin/pemetrexed; 107m for pemetrexed), mOS was NR across all groups. The objective response rate (ORR) was 1/8 (12.5%) vs. 5/16 (31%), and the disease control rate (DCR) was 7/8 (87.5%) vs. 11/16 (69%) with carboplatin/pemetrexed and pemetrexed respectively.

**Discussion:**

Carboplatin/pemetrexed was well tolerated and not associated with statistically significant differences in PFS compared to pemetrexed monotherapy in our combined cohort or thymic carcinoma and thymoma subgroups. Carboplatin/pemetrexed demonstrated a numerically longer PFS with encouraging disease control rates particularly in patients with thymoma.

## 1 Introduction

Thymoma and thymic carcinoma are rare thoracic malignancies with distinct clinical and pathologic characteristics both arising in the anterior mediastinum. Thymic epithelial tumors account for 10% of anterior mediastinal masses identified in the United States in individuals over 20 years of age (1). The incidence of thymoma is estimated at between 0.13-0.32/100,000 individuals per year (2). The estimated incidence of thymic carcinoma is even lower at between 0.07-0.38/100,000 individuals per year (3). These malignancies are defined by histologic categorization by WHO classification, which was most recently updated in 2021. Surgical resection remains the standard of care for eligible patients with both thymoma and thymic carcinoma, but palliative intent systemic therapy is often necessary in the setting of recurrence, metastatic spread, or patient and anatomic factors that preclude surgery (2, 3). This is a frequent challenge as 68% of thymic carcinomas have regional or distant spread at time of diagnosis (4). Due to the rarity of these tumors, prospective randomized trials guiding optimal systemic treatment strategies are limited, and current therapeutic approaches are largely informed by retrospective analyses and extrapolation of regimens established in the treatment of patients with primary lung cancer.

Pemetrexed, a multi-targeted antifolate agent, has demonstrated activity in thymic malignancies and is a recommended systemic therapy option for patients with advanced, unresectable, or recurrent thymoma and thymic carcinoma (5–7). A prospective phase II single-arm study of patients with recurrent thymoma and thymic carcinoma in 2005 found that in a mixed population of 27 pre-treated patients, pemetrexed had an acceptable toxicity profile and signs of therapeutic activity (8). In this study, the median time to progression of the combined cohort was 45 weeks and all 4 patients with responses by RECIST assessment had thymoma histology (8). In practice, platinum-based chemotherapy, specifically carboplatin is frequently given in combination with pemetrexed, extrapolating from the treatment of other thoracic malignancies. However, there are no published data on the safety or effectiveness of this combination in the treatment of thymic malignancies. Using retrospective data, this analysis aims to generate data on the safety and clinical outcomes associated with the use of carboplatin plus pemetrexed compared to pemetrexed monotherapy in an understudied thymic malignancy specific population.

## 2 Methods

This was a single-center retrospective cohort study performed at the University of Pennsylvania’s Abramson Cancer Center. The study period was from January 1, 2006 to December 6, 2024 and included patients who began treatment prior to June 1, 2024 to allow for a minimum of 6 months of follow-up time in all patients. This start date was selected as the preliminary results from the phase II trial demonstrating the activity of pemetrexed in patients with thymic malignancies was first presented in 2006 (4). This data led to the uptake of pemetrexed in clinical practice, starting in 2006 with few practice changing developments in palliative intent systemic therapy arising since that time. Cases were identified through the institution’s electronic health record filtering by the International Classification of Diseases, Tenth Revision (ICD-10) code for “malignant neoplasm of the thymus” (C37) and prior chemotherapy orders for pemetrexed. Charts of identified cases were manually reviewed to confirm a diagnosis of unresectable, recurrent, or metastatic thymoma or thymic carcinoma, and receipt of a pemetrexed-containing treatment regimen in any line of therapy. Demographic and clinical data were collected on all patients in the cohort. Patient characteristics were summarized by treatment group. Radiographic images were reviewed by investigators and time to disease progression and objective response were determined using Response Evaluation Criteria in Solid Tumors (RECIST) 1.1. Adverse events were retrospectively abstracted from clinical documentation and graded using the Common Terminology Criteria for Adverse Events (CTCAE) version 5.0.

Median progression-free survival (mPFS) and median overall survival (mOS) were estimated using Kaplan-Meier methodology and compared using the log-rank test. mPFS and mOS were measured from an index date of pemetrexed-containing regimen line of therapy initiation to radiographic or clinical progression or death. All statistical analyses were conducted using Stata v18.0 (StataCorp LP, College Station, TX, USA). Data were stored in a de-identified fashion on a HIPAA-compliant web-based application for data collection (REDCap v15.0.8, Vanderbilt University). This study was approved by the University of Pennsylvania’s Institutional Review Board (protocol #: 859981).

## 3 Results

### 3.1 Patient characteristics

A preliminary query of the electronic medical record identified 44 patients who were reviewed for eligibility. Of those 44 patients, 9 were excluded for not having a biopsy confirmed diagnosis of thymoma or thymic carcinoma. Another 5 patients were excluded because they had received pemetrexed-based chemotherapy in the neoadjuvant setting or concurrently with radiation, precluding consistent assessment of radiographic response and progression. A further 6 patients were excluded because their radiographic images were not available for review, resulting in a final cohort of 24 patients (see **Supplementary Figure 1**).

The overall cohort included 14 patients with thymoma and 10 with thymic carcinoma. The median age was 60 and 46% were male. The majority of patients self-identified as White (54%), 33% were Black, and 13% were Asian. The histologic subtype by WHO classification of thymic tumors in this population was notable for two patients (8%) with class AB, three patients (13%) with class B1, seven patients (29%) with class B2, and two patients (8%) with class B3-C thymoma and 10/24 (42%) patients with thymic carcinomas. Classification was based on 3^rd^ edition criteria in 10/24 cases (42%) and 4^th^ and 5^th^ edition in 14/24 cases (58%). The cohort included both patients with recurrent disease (46%) and *de novo* metastatic disease (54%). Most patients had previously received surgery (71%) and radiation (75%).

A total of 8 patients received carboplatin/pemetrexed (5 thymoma, 3 thymic carcinoma) for a median of 12 cycles (which consisted of a median of 4 cycles of carboplatin with a range of 4–7 cycles followed by pemetrexed maintenance until progression. A total of 16 patients received pemetrexed alone (9 thymoma, 7 thymic carcinoma) for a median of 8 cycles. Patients in the carboplatin/pemetrexed group were younger than those in the pemetrexed group with a median age of 56 vs. 61 years. Of the 8 patients who received carboplatin/pemetrexed, 5 (62.5%) received the regimen as a first-line systemic therapy and 3 (37.5%) had received prior platinum-based chemotherapy. The previous platinum-based chemotherapy received was cyclophosphamide, doxorubicin, and cisplatin (CAP) in 1 case as first-line therapy and carboplatin/paclitaxel as first-line in 1 case and fourth-line therapy in 1 case. Of the 16 patients treated in the pemetrexed monotherapy treatment arm, pemetrexed was administered as a first-line systemic therapy in 3/16 (19%) of patients vs. second-line and beyond in 13/16 (81%) patients. With the exception of one patient who declined platinum-based chemotherapy, all patients in the pemetrexed arm received platinum-based chemotherapy as either neoadjuvant, radiosensitizing, or salvage systemic therapy.

In patients with thymoma, carboplatin/pemetrexed was more commonly administered in the first line setting (n=4, 80%). Among patients with thymic carcinoma, carboplatin/pemetrexed was most often given in the 4th line or later setting (n=2, 67%). There were no significant differences between the treatment groups in terms of baseline Eastern Cooperative Oncology Group (ECOG) performance status, prior cancer-directed therapies, or demographic characteristics (**Table 1**).

**Table 1:** Patient characteristics.

	Carboplatin/ pemetrexed n=8	Pemetrexed n=16
Diagnosis		
Thymoma	5 (63%)	9 (56%)
Thymic carcinoma	3 (38%)	7 (44%)
Median age	56 (range 31-78)	61 (range 27-82)
ECOG		
Thymoma		
0	2 (40%)	3 (33%)
1	1 (20%)	6 (67%)
2	1 (20%)	0
3	1 (20%)	0
Thymic carcinoma		
0	1 (33%)	3 (43%)
1	1 (33%)	4 (57%)
2	1 (33%)	0
Sex, n (%)		
Male	3 (38%)	8 (50%)
Female	5 (62.5%)	8 (50%)
Race		
White	4 (50%)	9 (56%)
Black	4 (50%)	4 (25%)
Asian	0	3 (19%)
Stage		
I-III, recurrent	4 (50%)	7 (44%)
IVA	3 (38%)	6 (38%)
IVB	1 (13%)	3 (19%)
History of surgery		
Yes	6 (75%)	11 (69%)
No	2 (25%)	5 (31%)
Resection		
R0	2 (25%)	0
R1	3 (38%)	4 (25%)
R2	1 (12%)	3 (19%)
Unknown	0	4 (25%)
No Resection	2 (25%)	5 (31%)
History of radiation		
Yes	6 (75%)	12 (75%)
No	2 (25%)	4 (25%)
Line of systemic therapy		
Thymoma		
1	4 (80%)	1 (11%)
2	1 (20%)	6 (67%)
3	0	2 (22%)
Thymic carcinoma		
1	1 (33%)	2 (29%)
2	0	1 (14%)
3	0	3 (43%)
4+	2 (67%)	1 (14%)

### 3.2 Outcomes

**Figure 1:**
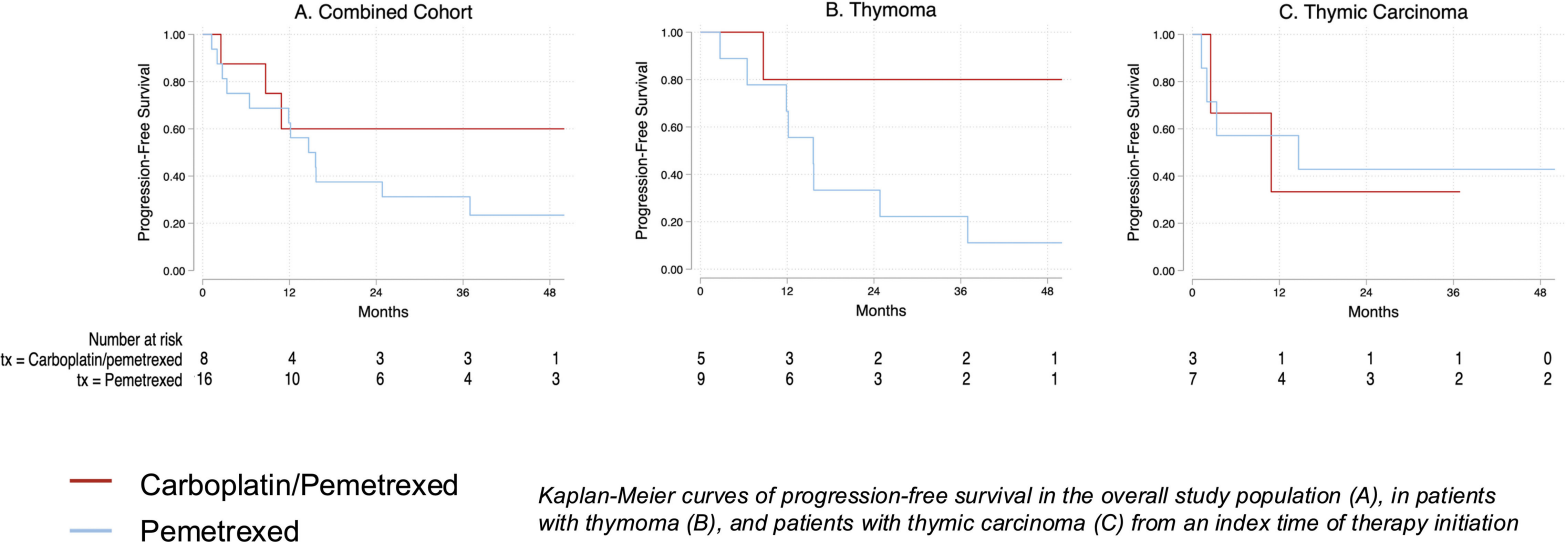
Kaplan-Meier graphs representing progression-free survival in the overall study cohort and thymoma and thymic carcinoma subgroups.

**Figure 2:**
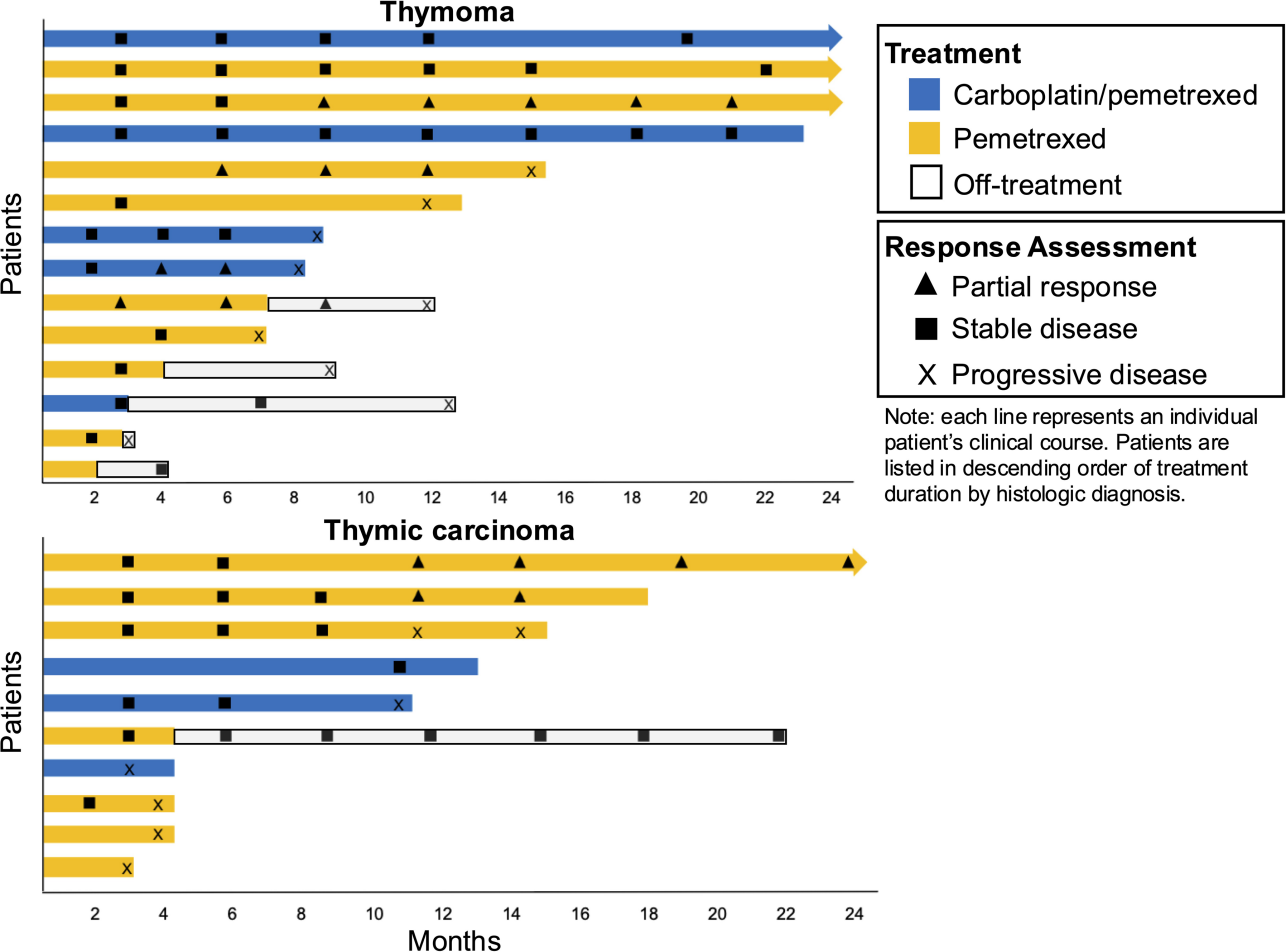
Swimmer plots showing duration of therapy in months with disease response. Response assessments indicated by symbol as either partial response (triangle), stable disease (square), or progressive disease (X) at specific timepoint.

Median PFS was not reached in the overall carboplatin/pemetrexed group vs. 14.7 months in the pemetrexed monotherapy group (p=0.27). In the combined cohort at a median follow-up duration of 37 months in the carboplatin/pemetrexed group and 107 months in the pemetrexed group, mOS was not reached in either treatment group. Among 14 patients with thymoma, mPFS was NR with carboplatin/pemetrexed (n=5) vs. 15.6 months with pemetrexed (n=9) (p=0.09). Among 10 patients with thymic carcinoma, mPFS was 10.9 months with carboplatin/pemetrexed (n=3) vs. 14.7 months with pemetrexed alone (n=7) (p=0.88) (see **Figure 1**). The objective response rate (ORR) was 1/8 (12.5%) with carboplatin/pemetrexed and 5/16 (31%) with pemetrexed. The partial response with carboplatin/pemetrexed occurred in a patient with thymoma while with pemetrexed, three partial responses occurred in patients with thymoma and two in patients with thymic carcinoma. The disease control rate (DCR) was 7/8 (88%) with carboplatin/pemetrexed with 5/5 (100%) patients with thymoma and 2/3 (67%) patients with thymic carcinoma experiencing disease control. The DCR rate with pemetrexed was 11/16 (69%) with 7/9 (78%) patients with thymoma and 4/7 (57%) patients with thymic carcinoma experiencing disease control (see **Figure 2**).

### 3.3 Safety

A total of 6 patients (75%) receiving carboplatin/pemetrexed experienced an adverse event (AE) of any grade while undergoing treatment compared to 11 patients (69%) in the pemetrexed group. Most AEs in both groups were grade 1 or 2, with just 1 patient (12.5%) in the carboplatin/pemetrexed group and 3 patients (19%) in the pemetrexed group experiencing a grade 3 AE. There were no grade 4+ events in either group. Hematologic toxicity was the most common AE in both groups, affecting 5 patients (62.5%) in the carboplatin/pemetrexed group and 4 patients (44%) in the pemetrexed group (see **Table 2**). No patients in the carboplatin/pemetrexed group required dose-reductions due to adverse events, while 3 (19%) patients in the pemetrexed group required dose reductions.

**Table 2:** Safety.

	Carboplatin/ pemetrexed n=8	Pemetrexed n=16
Required dose reduction?	0	3 (18.8%)
Hematologic toxicity		
Any Grade	5 (62.5%)	7 (43.8%)
Grade 1	2 (25%)	6 (37.6%)
Grade 2	2 (25%)	–
Grade 3	1 (12.5%)	1 (6.3%)
Dermatologic toxicity		
Any Grade	–	4 (25%)
Grade 1	–	2 (12.5%)
Grade 2	–	1 (6.3%)
Grade 3	–	1 (6.3%)
Other toxicity		
Any Grade	4 (50%)	4 (25%)
Grade 1	–	1 (6.3%)
Grade 2	3 (37.5%)	1 (6.3%)
Grade 3	1 (12.5%)	2 (12.5%)

## 4 Discussion

In this retrospective analysis, the combination of carboplatin and pemetrexed was well tolerated and associated with high rates of disease control. However, it was not associated with a high rate of response (12.5%) and did not demonstrate a statistically significant improvement in PFS compared to pemetrexed monotherapy. Among patients with thymoma, the combination was associated with a numerically higher mPFS (NR vs. 15.6 months, p=0.09), suggesting further investigation may be warranted, particularly among patients treated in an earlier line of therapy. However, it must be noted that in our subgroup of thymoma patients, individuals treated with carboplatin/pemetrexed were more often treated in the first-line setting. As a result of this potential confounder, larger multi-center studies are needed to demonstrate the effectiveness of carboplatin/pemetrexed combinations in patients with thymic malignancies. We note that in this retrospective, non-randomized study, physician selection of treatment with carboplatin/pemetrexed vs. pemetrexed alone, which was influenced by patient comorbidities, overall performance status, and prior response to platinum-based chemotherapy may have influenced outcomes. Further, given the decreased follow-up time in the carboplatin/pemetrexed group (37 months) as compared to the pemetrexed monotherapy group (107 months), OS data are considered immature. This differential follow-up may impact the reliability of OS comparisons in this cohort and as such, mPFS is considered a more reliable endpoint for our effectiveness analysis. Interestingly, in our sample, pemetrexed demonstrated more activity in thymic carcinoma than expected based on prior studies and despite the squamous histologic features of these tumors (3, 5). Additionally, our study, only included patients who began treatment prior to June 2024, so we were not able to assess real-world outcomes or generate comparisons between carboplatin/pemetrexed and regimens based on trials published after that date. Specifically, our sample did not include any patients treated with carboplatin, paclitaxel, and ramucirumab, which demonstrated promising response rates in a treatment-naïve thymic carcinoma population in the single-arm phase II RELEVENT trial published in September 2024 (9).

Importantly, carboplatin/pemetrexed may represent a less toxic potential alternative to more intensive multi-agent regimens such as CAP, which remains a commonly used first-line therapy in advanced thymoma and thymic carcinoma. Although CAP has demonstrated high response rates of approximately 50% in previous studies, its use is often limited by toxicity, particularly in older adults or those with comorbidities (1). Other platinum-based regimens such as carboplatin and paclitaxel, demonstrated an objective response rate of 42.9% in patients with thymoma (mPFS = 16.7 months) and 21.7% in individuals with thymic carcinoma (mPFS = 5 months) (10). The addition of ramucirumab to this regimen in a single-arm trial of treatment-naïve patients with thymic carcinoma led to an objective response rate of 80% and mPFS of 18.1 months (9). These outcomes were achieved largely in patients with robust ECOG PS and limited prior therapy and notably were associated with significant rates of grade >3 cytopenias and neuropathy (9, 10). By contrast, in this cohort, which included many heavily pre-treated patients, carboplatin/pemetrexed was associated with a favorable toxicity profile, with manageable hematologic side effects, and low rates of severe non-hematologic toxicity. However, given the study’s small sample size, extended duration, and reliance on querying older electronic medical record data, we acknowledge that compared to prospective trials, this study may have decreased sensitivity for detecting adverse events. The tolerability of this regimen should be further studied in more contemporary multicenter settings.

While the response rate of carboplatin/pemetrexed in this study was only 12.5%, the combined regimen was associated with a high rate of disease control at 87.5%. As such, carboplatin/pemetrexed may be considered for patients who are ineligible for anthracycline-based chemotherapy or are undergoing palliative-intent treatment wherein toxicity mitigation and quality of life are prioritized. This appears to be occurring in actual clinical practice, as several of the patients in this cohort treated with first-line carboplatin/pemetrexed were selected for this treatment due to physician concerns for general frailty, disease-specific factors, or specific comorbidities. In detailed chart review of cases, where carboplatin/pemetrexed was utilized in the absence of prior CAP or carboplatin/paclitaxel exposure, treating physicians noted patients had pre-existing clinically significant neuropathy, cardiac co-morbidities, patient preferences to avoid certain toxicities, or a more indolent disease course, which influenced their decision to preferentially utilize carboplatin/pemetrexed.

Future research using data from multiple centers is needed to confirm our findings and determine which patients are likely to derive benefit from this combination. Ideally, future multi-center prospective studies can directly compare carboplatin/pemetrexed to other multi-agent regimens used in the treatment of thymic malignancies, such as carboplatin/paclitaxel and CAP and inform optimal treatment sequencing. Despite the limitations of this study’s retrospective design, small sample size, variable histology, and follow-up between treatment arms, our findings provide necessary real-world safety data informing the use of carboplatin/pemetrexed for patients with these rare thoracic malignancies.

## Data Availability

The data analyzed in this study is subject to the following licenses/restrictions: single-institution, retrospective data is subject to institutional restrictions. Individual-level data are not made available to protect patient confidentiality. Requests to access these datasets should be directed to BB, benjamin.bleiberg@pennmedicine.upenn.edu.

## Supplementary material

Supplementary Figure 1 CONSORT diagram.
