# Supplementary figures and images for: Carboplatin plus pemetrexed versus pemetrexed alone in advanced thymoma and thymic carcinoma: a retrospective cohort study
Source: Front Oncol. 2026 Apr 22;16:1795920. doi: 10.3389/fonc.2026.1795920 (PMC13143526; doi:10.3389/fonc.2026.1795920)

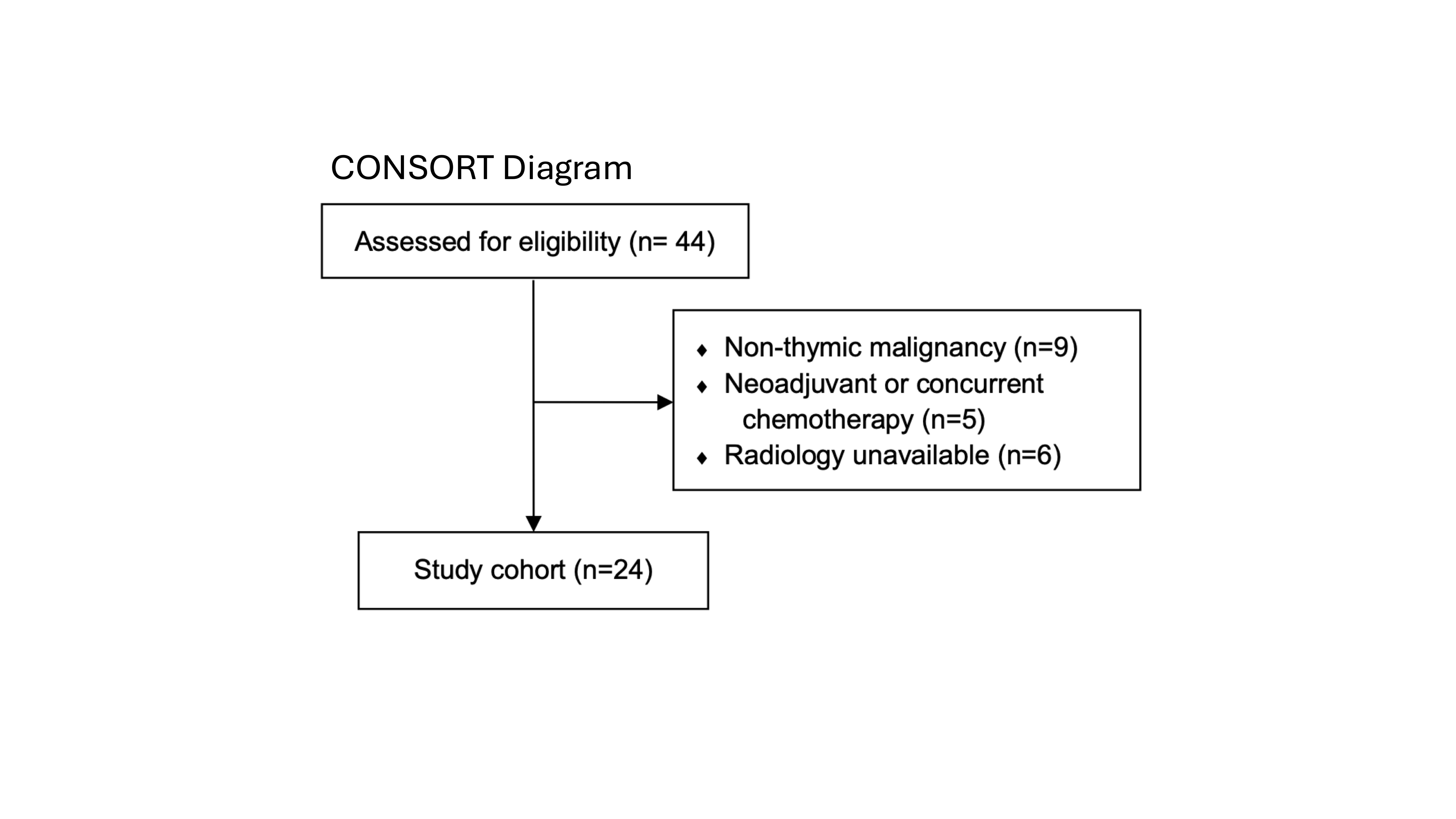

Supplement: Supplementary Figure 1 — CONSORT diagram. [file Image1.jpeg]
